# On the Morbid Conditions of the Blood

**Published:** 1847-02

**Authors:** Geo. N. Burwell

**Affiliations:** Buffalo


ART. II.—*On the morbid conditions of the Blood.* By Geo. N. Burwell, M. D.

[Dr. Flint.—An apology may perhaps be due to you, and to your city readers for my sending for your Journal as the original article promised to you by me for this month, a paper read before the Erie County Medical Society at its late meeting. Not having had leisure to fulfil both obligations separately, I was under the necessity of meeting them conjointly.]

Yours very truly,

G. N. BURWELL.

---

The subject, Gentlemen of the Society, upon which I propose to address you, is a brief notice of some of the Diseases of the Blood. The medical mind, after swaying like a mighty pendulum from solidism to humoralism, and from humoralism to solidism, is now again verging towards humoral doctrines less exclusive and more correct, as the data upon which they are founded partake of the nature of facts, rather than of crude notions of peccant humors, of coction, etc.

The first cultivators of medicine, with only a rude idea of anatomy and physiology, could only judge of disease by its symptoms during life, and the traces of it discoverable upon the solids after death; and their treatment was exclusively directed to the administration of remedies for the relief of this or that symptom, a system of practice too much in vogue, I am afraid, even at this day.

But after the discovery of the circulation of the blood, and after the application of the microscope to the examination of those changes in the blood so evident, in many diseases, to the unassisted eye even, an exclusive humoral pathology, as applied to almost all diseases, began gradually to be adopted; and which, like most new theories, fascinating by its apparent truth and simplicity, and formed without sufficient investigation, was full of error, and led to a correspondingly evil result when applied to practice. This was the period of concoction of the liquors of the Body, of acid humour, and offending spirits.

The period of reaction at length arrived, and exclusive solidism again became the test of medical truth and pathology. Inflammation was the sole "fons et origo" of all diseases, and the antiphlogistic regimen the grand panacea for all the ills that flesh is heir to. The lancet, leeches, and iced water took the place of hot, stimulatating medicines and drinks, and the "diete absolue" of the comfortable meals heretofore furnished to invalids.

But time, the great test of all tiuth, shewed after awhile that this treatment, founded upon the presumed inflammatory element of all diseases, did not succeed as well as anticipated, although much better than the one it took the place of. This theory is now going to the shades of the past, accompanied, as if in a funereal procession, by some of its early advocates, who resist the innovations of time, and prefer, if such must be its destiny, to accompany it to to the tomb, themselves its sole representatives and mourners.

We are now about past the reaction from Broussaism, and the medical mind is in an inchoative stage, waiting for the developments of experiment and observation, and forming opinions only as warranted by facts, thrice proved, by experience, by the microscope, and by organic chemistry.

The blood is the great and important organizable fluid of the body. It is liquid flesh; the material from which all structures and organs are evolved, for it contains within itself the whole substance of the body. Its principle function, therefore, is to supply nutritive matter for the growth and reparation of all parts of the system. Collateral with this, is its office to furnish all the secretions necessary for the proper performance of the various functions; and also its office as a carrier to the various excretory organs of those compounds formed in the metamorphosis of the tissues, and destined to be eliminated from the organism. It is by an act of life, peculiar to itself, that each organ exercises a power of selection from the various ingredients in the blood of those parts only which are adapted to its nutrition, and for its secretion. The nature of this vital act, its source, and the laws which control its action, are probably, among the ultimate facts of life which will forever remain hidden from human knowledge and investigation.

The blood is a very complex fluid, being composed, according to the best chemical analyses of no less than twenty-six different substances; besides having oxygen, nitrogen, and carbonic acid gas mixed with it, the same as these gasses are contained in all water from which they have not been expelled by a special process.

Besides being of so compound a nature, it is undergoing incessant changes in its course through the capillaries of the system. Indeed, we are constantly speaking of four different kinds of blood, the arterial, the venous, the portal and the menstrual.

To the naked eye, the blood as it runs from a divided vein appears to be a homogenious fluid, but, by its coagulation, the following are shown to be the principal parts that enter into its composition; 1st, Red corpuscles or the hematozin, 2d, Fibrin; 3d, the Serum, containing numerous animal matters and inorganic salts, with water as its base. All these ingredients, of course bear certain proportions to each other, which it is not my purpose here to give. Variation from these proportions, beyond certain limits which have been proved to be compatible with health, constitutes one form of disease or disorder of the blood.

By means of the microscope we are enabled to make a farther analysis, not of the chemical elements of dead blood, but of living organic structures, floating in, and constituting a part of the blood. The most important of these are the red-corpuscles, or globules, as they are also called, but which are shown to be flattened cell-discs. Their office, it is pretty well settled, is to carry oxygen from the lungs to all parts of the system, which is there exchanged for carbonic acid, to be brought to the lungs for elimination.

Second, there are the white corpuscules; and third, there are minute granules or spherules of matter, supposed to be albuminoid. The functions or offices intended to be fulfilled by these particles are not yet settled.

Such being the chemical and organic constitution of the blood, it can readily be comprehended what a constant tendency there must be to change; what a variety of affinities exist within it, each operating against the other; and what a wonderful power that vital force must be which keeps up that harmonious action so necessary to the existence of life and health. Indeed, upon a moments reflection, it is more a matter of astonishment that life exists so long, opposed as it is by so many forces from within, and daily trifled with by the thoughtless votary of passion, regardless of everything, but the momentary gratification of his desires.

There are two great and distinctive classes of alterations of the blood in disease. The first, and the one to which I shall principally confine my remarks, are those which originate in the blood itself. The second are those disorders secondary to organic disease.

There are two principal divisions of the first class, the innate or hereditary (congenital as we would say of organic disease,) and the acquired. The acquired may be farther divided into the acute and the chronic. The innate or congenital diseases of the blood are almost universally chronic; the only exception that occurs to me at present, being those rare cases, where a child is born with variola.

In these general divisions, it is seen how closely are humoral diseases susceptible of division into the same classes into which we are accustomed to divide organic diseases.

Under the head of congenital diseases of the blood, I shall first place those constitutional diseases, or states of the system predisposing to disease commonly known under the name of *the Diatheses*. They are the Scrofulous, Tubercular, Gouty, Rheumatic, Hemorrhagic, Syphilitic and Cancerous diatheses. I wouldalso include under the same head, the following diseases generally classified as Renal; viz: the Oxalic, Lithic and Phosphatic diatheses, some cases of Bright's disease of the kidney, and the different forms of Diabetes, all of which are clearly constitutional diseases, and oftentimes indubitably hereditary.

What the particular affection of the blood is, that distinguishes these diatheses, we have not yet been able to discover. The hereditary tendency of all of them, the numerous organs and distinct tissues they simultaneously attack, prove their constitutional character. Their seat, evidently, is primarily in the blood, although they destroy by superinduced organic disease. Their pathological element is probably some change of character, or an hereditary want or perversion of the vital force which governs the changes constantly going on in the blood.

The acquired diseases, of an acute character, may be sub-divided into four classes, as follows:

*The first class.* Those of contagious origin, which includes the Typhus or spotted fever, Typhoid fever, the Exanthemata, Pertussis, Malignant Pustule, Glanders, Wounds from dissection, Syphilis, Hydrophobia, and probably some varieties of the epidemic Erisypelas and puerperal fever, so prevalent of late years in different portions of our country. These are all propagated by what are commonly called, animal poisons.

*The second class.* Those arising from vegetable poisons or miasma (if such an element be allowed to exist). These comprise the almost endless varieties of Intermittent, Remittent and yellow-fever; and perhaps also the Plague of Egypt and the Mediteranean.

*The third class.* Those arising from epidemic causes. Asiatic cholera and Influenza are the types of this class.

*The fourth class.* Those arising from some special cause; as 1st. the destruction of the coagulability of the blood (and consequent death) from a stroke of lightning; from breathing noxious vapors; or from continued over-exertion, as sometimes seen in animals hunted to death. 2d.—That condition of the blood seen in Scurvy.

There are three diseases placed in this class of acute-acquired, which by many may not be considered as of humoral origin. They are Hydrophobia, Pertussis and Influenza. I put them where they are, from their great analogy to the diseases with which they are classified. The nervous element certainly is apparently better marked in them than any humoral element; yet I feel confident, that future investigation will show the propriety of their classification with their congeners.

The enumeration of acquired diseases of a chronic character will include many already put under other heads. This is especially the case with those classified as innate or hereditary. All of them may be acquired, and exist in a chronic form; that is, they are not, in every case, necessarily congenital. The only acute acquired diseases which may become chronic, are Syphilis in some of its various forms, Intermittent fever and Scurvy.

The following diseases belong exclusivly to this class—1st, Plethora; 2d, many cases of chronic Anemia (including chlorosis); 3d. Melanosis; 4th, some chronic diseases of the Skin. I may here remark that many diseases of the skin are undoubtedly closely connected, if not identical, with many of the diatheses already mentioned, and exist as one form of their manifestation. We already know this to be the case in the Exanthemata and in Syphilis. Farther researches will without doubt show the same result in the Tubercular and other diatheses.

Under a *fifth* head, I would include a set of cases of evident disease of the blood, which are often met with in practice, and task to the utmost, the skill and resources of the physician. They often terminate in death, sometimes suddenly and unexpectedly to all. Their accession is slow, and their duration often tedious and protracted. The prognosis is always bad in confirmed cases.

When called to see them, they complain principally of general feebleness and ill health. We perhaps find them sitting up, and are told that they keep up a general supervision of their affairs, although not able to do much of themselves. They are, according to my experience, very generally married women. They are soon to be confined, or already have been confined some time before; had suffered a good deal before their confinement, and had a slow getting up. With some, lactation brings on this state of things; other cases have neither of these conditions to refer to as a cause.

There is general emaciation; they are, perhaps, nervous and restless; their complexion is pale and waxen, or more frequently, it has a sallow, icterode hue. There is some tumidity of the face, which may be constant, or only noticed at intervals; their ankles are often œdematous; sometimes there is universal anasarca. Their lips are bloodless and dry. The state of the tongue varies much in different cases; it may be morbidly red and smooth, or, on the contrary, pale, flabby and loaded with a pasty fur. There may or may not be ulcerations of the tongue, lips, or gums. Sometimes there is excessive sputation, which in one case I have known, was complained of as being uncomfortably warm. The bowels may be regular, with healthy, although scanty, discharges; or there may be alternations of diarrhœa and constipation, with tympanitis. The appetite is variable, being sometimes natural or voracious even, and again so entirely gone, that the nicest articles of food only excite loathing and disgust; there is often thirst and some traces of feverishness, with a pulse quick, and at 85 to 100 beats in a minute. We are told that the urine very commonly becomes heavy on cooling; that is, deposits a sediment of the yellow or red lithates.

The patient is apt to complain much of oppression at the chest, of dyspnoea on any exertion, with palpitation, or, as it is more frequently called, fluttering at the heart. This fluttering is often accompanied with turns of great, and to the patient of alarming faintness.

A cough with some mucoid expectoration, and a pain in the left side often complained of as being very sharp, makes us fear Phthisis, and induces a thorough physical examination of the chest, but which is unsatisfactory so far, as in not finding disease to account for the symptoms.

There may be profuse and too frequently recurring turns of menstruation, with leucorhœa in the intervals. Again, on the contrary, the menses may be very scanty.

In some cases we find the skin harsh and dry, and subject to eruptions especially of the dry scaly varieties. Again it is relaxed and moist, and the patient is subject to free perspirations at night. Their sleep is sometimes described as being good, or they may complain of never feeling sleepy, but will lie awake nearly all the night, perfectly easy and free from any pain or other source of anxiety. Headache is a prominent symptom with some.

After a prolonged and attentive examination, we do not find any organic disease to account for the symptoms. Relying on this, we are perhaps too forward in promising a cure. He who does it too hastily will often have cause to repent of his want of caution. We succeed, indeed, in curing many cases; but, in others, where we most expected to cure, we find all our remedies to fail in arousing the sinking energies of the system. The feebleness becomes greater and greater, a troublesome cough, or other distressing symptom continues to the last, until the system yields to the great conqueror, Death.

In other cases, syncope suddenly sets in while the patient is making some exertion to get up, or to move from one chair to another, or to the bed. She cries out, she is fainting, and falls down, dead. Or a state of stupor comes on, which, in a few days, passes into confirmed coma and death; or instead of stupor, the friends are alarmed by the patient's becoming suddenly talkative and incoherent, and rapidly relapsing into furious delirium or insanity. After passing a few days in this state, with intervals, perhaps, of partial lucidness, she gradually ceases to talk, it soon becomes difficult to rouse her, and she dies comatose;—or instead of this her breathing becomes rapid, expectoration difficult, and she dies from congestion of the lungs.

A post-mortem examination fails to find any tubercles of the lungs, or diseased heart, or liver, or kidneys, to account for death. We are forced to refer the cause to some badly understood disease of the circulating mass.

This concludes the classification of diseases or disorders of the blood of the first class. I shall next briefly enumerate these changes secondary to organic disease.

The *first*, is an increase, far beyond the normal proportion, of the fibrine in the blood as the result of a state of inflammation of any organ or tissue of the body. This is a discovery of late years, and one having a very important bearing upon pathology and practice.

2d. Anemia, a condition, the common consequence of chronic disease of any of the organs essential to life, especially those of the lungs, stomach, liver and kidneys. It does not necessarily follow as a direct consequence of disease of the brain or heart. I would also include under this head, those cases of anemia from hemorrhage secondary to wounds, or to miscarriage or labor.

3d, Disorder of blood from a retention of bile, resulting from an hindrance to the flow of this fluid through the ducts of the liver, or from a want of secretion from inflammation, congestion or other obstruction of this organ.

4th, Poisoning of the blood by retention of the elements of the urinary secretion, from similar diseases of the kidneys or their excretary ducts.

5th, Cases of Cyanosis from congenital malformation of the heart, or of the large blood-vessels,

6th, Disease of the blood from purulent absorption.

*Of the Causes of Disorders of the Blood.*—The *first* in the list is to be found in the nature of the diseases themselves; that is, their capability of transmission from one generation to another. This is especially the case with all the diseases heretofore classified as innate or congenital.

The *second* cause is found in the peculiar nature of the diseases which we call contagious, miasmatic or epidemic. In what these properties cousist we do not know. They probably reach the blood by introduction into the lungs with the air we inhale, and apparently act as a ferment, producing changes in the blood which are accompanied with symptoms different with each of the different poisons. This is especially the case with the exanthemata, and the miasmatic and epidemic diseases.—Syphilis, malignant pustule, and glanders are not thus communicable. They require to be inoculated into the system before they produce their regular symptoms.

The *third* cause is found in a retention of the secretions; their non elimination from the blood and discharge from the body. The lungs, the liver, the kidneys and the skin are the four principal depurating organs of the body. All must properly perform their functions for the blood to remain healthy. When one is deranged the others suffer more or less, and the whole function of nutrition becomes immediately disordered from the circulation of impure blood.

The *fourth* source of diseased blood is found in the introduction into this fluid of too much nutritive material, or of matters incapable of being applied to the nutrition of the body. There is no system so uniformly abused as the digestive system. We eat too rapidly, too much, and of too great a variety of dishes at once. We also daily drink too much and too strong tea and coffee and, perhaps spirituous liquors. The blood is overloaded with nutritive matters, which oppress all the organs and interfere with the due and regular performance of the important function of nutrition. Hunger and thirst were given to us that we should not neglect to supply the organism with material to repair the daily waste. We have elevated them to be our Gods, and sacrifice to them our health, reputation, all the daily comforts springing from temperance and sobriety, and finally our life itself. Obedience to such passions is not without serious consequences. Indisposition or inability for exertion, listlessness or actual depravation of the mind, dyspeptic complaints, serious humoral disorders as gout, and the different urinary diatheses, and organic disease of the stomach, liver, or kidneys, are the daily consequences of intemperance and gluttony.

*Fifth.* The state of inflammation, some organic diseases of the heart, obstructions of the liver and kidneys, collections of matter in the system are frequent causes of alteration in the blood.

*Sixth.* Lightning, over-exertion and noxious vapours have the power of destroying the vitality of the blood.

*Seventh.* Besides the above, there are unknown causes of humoral disease; for instance, we do not know the cause of melanosis, nor of many other states of evident disorder of the blood,

I shall conclude by giving a brief summary of the general laws or facts relating to humoral diseases.

1st. All the congenital diseases of the Blood are of the chronic form, except variola of the new-born child.

2d. All these congenital diseases are hereditary; and no other disease of the human body is hereditary, except some forms of functional nervous disorders. I refer particularly to insanity and to some varieties of neuralgia. This is a very curious fact, and suggests reflections respecting the nature of these two very distinct classes of disease, and the cause of such an analogy in one respect only.

3d. All the contagious, miasmatic and epidemic diseases, except hereditary cancer and hereditary syphilis, are in all cases acquired diseases of the blood, and with the same exceptions, none of them are hereditary. This is another curious and interesting fact.

4th. All the acute acquired diseases, except syphilis, have a rapid course and generally soon terminate one way or the other.

5th. They often destroy without sufficient organic disease being found to account for death; although in all of them there is a constant tendency to disease of some of the organs of the body.

6th. They often terminate by the occurrence of some other form of disease of the blood; more especially in scrofula and phthisis.

7th. The chronic acquired and hereditary diseases of the blood may pursue a rapid or a protracted course, according to the favorable or unfavorable circumstances by which the patient is surrounded.

8th. They are all very liable to attack many organs and different tissues of the system, at once, or in rapid succession; thus becoming complicated with organic disease.

9th. The mode in which phthisis, scrofula and cancer thus develop secondary organic disease is by the deposit or diffusion through one or more organs, of matter either generated anew in the blood, or of matter already there, which has not undergone the requisite changes to convert it into wholesome nutriment for the system.

This deposit, like any other foreign matter, either excites inflammation, or otherwise interferes with the functions and nutrition of the organ or organs in which it may be deposited.

10th. The hemorrhgic, rheumatic, gouty and urinary diatheses have not so constant a tendency to the development of organic disease, as the diatheses just above named; although they frequently are thus complicated.

11th. In the large majority of cases of death from original humoral disease, the immediate cause of dissolution is the organic disease previously excited; and which is often erroneously supposed to be the only cause.

I have thus given a meagre and skeleton-like outline of the composition of the blood and its diseases and their classsification, their causes, and some of the general laws of their course, complications and terminations. It would require a volume instead of a hasty sketch of this sort to do justice to the subject.

*Buffalo, January*, 1847.

